# Combination treatment with recombinant methioninase enables temozolomide to arrest a BRAF V600E melanoma in a patient-derived orthotopic xenograft (PDOX) mouse model

**DOI:** 10.18632/oncotarget.20231

**Published:** 2017-08-12

**Authors:** Kei Kawaguchi, Kentaro Igarashi, Shukuan Li, Qinghong Han, Yuying Tan, Tasuku Kiyuna, Kentaro Miyake, Takashi Murakami, Bartosz Chmielowski, Scott D. Nelson, Tara A. Russell, Sarah M. Dry, Yunfeng Li, Michiaki Unno, Fritz C. Eilber, Robert M. Hoffman

**Affiliations:** ^1^ AntiCancer, Inc., San Diego, CA, USA; ^2^ Department of Surgery, University of California, San Diego, CA, USA; ^3^ Department of Surgery, Graduate School of Medicine, Tohoku University, Sendai, Japan; ^4^ Division of Hematology-Oncology, University of California, Los Angeles, CA, USA; ^5^ Department of Pathology, University of California, Los Angeles, CA, USA; ^6^ Division of Surgical Oncology, University of California, Los Angeles, CA, USA

**Keywords:** recombinant methioninase, methionine dependence, metabolic targeting, temozolomide, melanoma

## Abstract

An excessive requirement for methionine termed methionine dependence, appears to be a general metabolic defect in cancer. We have previously shown that cancer-cell growth can be selectively arrested by methionine deprivation such as with recombinant methioninase (rMETase). The present study used a previously-established patient-derived orthotopic xenograft (PDOX) nude mouse model of BRAF V600E-mutant melanoma to determine the efficacy of rMETase in combination with a first-line melanoma drug, temozolomide (TEM). In the present study 40 melanoma PDOX mouse models were randomized into four groups of 10 mice each: untreated control (n=10); TEM (25 mg/kg, oral 14 consecutive days, n=10); rMETase (100 units, intraperitoneal 14 consecutive days, n=10); combination TEM + rMETase (TEM: 25 mg/kg, oral rMETase: 100 units, intraperitoneal 14 consecutive days, n=10). All treatments inhibited tumor growth compared to untreated control (TEM: *p*=0.0081, rMETase: *p*=0.0037, TEM-rMETase: *p*=0.0024) on day 14 after initiation. However, the combination therapy of TEM and rMETase was significantly more efficacious than either mono-therapy (TEM: *p*=0.0051, rMETase: *p*=0.0051). The present study is the first demonstrating the efficacy of rMETase combination therapy in a PDOX model, suggesting potential clinical development, especially in recalcitrant cancers such as melanoma, where rMETase may enhance first-line therapy.

## INTRODUCTION

Melanoma becomes a recalcitrant cancer when it metastasizes to regional lymph nodes, with a 5-year survival rate of 29%; and 7% when it metastasizes to organs [1]. There is still no cure for stage III and IV melanoma due to drug resistance, tumor heterogeneity and an immune-suppressed tumor microenvironment [1-5]. Temozolomide (TEM), an alkylating agent, is first-line chemotherapy for melanoma but with limited efficacy [1-5]. Targeted chemotherapy and immuno-therapy are also of limited efficacy in melanoma [1-5]. Melanin may also interfere with therapy [4,5]. Therefore, more effective approaches to melanoma treatment are needed.

Toward the goal of precision personalized oncology, our laboratory pioneered the patient-derived orthotopic xenograft (PDOX) nude mouse model with the technique of surgical orthotopic implantation (SOI), including pancreatic [6-9], breast [10], ovarian [11], lung [12], cervical [13], colon [14-16], stomach [17], sarcoma [18-22] and melanoma [23-26].

Previously, a BRAF-V600E-mutant melanoma obtained from the right chest wall of a patient was transplanted orthotopically in the right chest wall of nude mice to establish a PDOX model [24-26]. Trametinib (TRA), an MEK inhibitor, caused tumor regression. In contrast, another MEK inhibitor, cobimetinib (COB) could slow but not arrest growth or cause regression of the melanoma PDOX. TEM could slow but not arrest tumor growth or cause regression [24].

Methionine dependence is a general metabolic defect in cancer. Methionine dependence is due to excess use of methionine for aberrant transmethylation reactions, termed the Hoffman effect, analogous to the Warburg effect for glucose in cancer [27-32]. The excessive and aberrant use of methionine in cancer is strongly observed in [^11^C] methionine PET imaging, where high uptake of [^11^C] methionine results in a very strong and selective tumor signal compared with normal tissue background. [^11^C] methionine is superior to [^18^C] fluorodeoxyglucose (FDG)-PET for PET imaging, suggesting methionine dependence is more tumor-specific than glucose dependence [33, 34].

A purified methionine cleaving enzyme, methioninase (METase), from *Pseudomonas putida* has been found previously to be an effective antitumor agent *in vitro* as well as *in vivo* [35-38]. For the large-scale production of METase, the gene from *P. putida* has been cloned in *Escherichia coli* and a purification protocol for recombinant METase (rMETase) has been established with high purity and low endotoxin [39-41].

It has been demonstrated that methionine deprivation arrests growth and induces a tumor-selective G_2_-phase cell-cycle arrest of cancer cells *in vitro* and *in vivo* [42-45].

MET depletion therapy, using rMETase, sensitized brain tumors to TEM in xenografts in nude mice [46].

We reported recently on the efficacy of rMETase against Ewing’s sarcoma in a PDOX model. rMETase effectively reduced tumor growth compared to untreated control. The methionine level both of plasma and supernatants derived from sonicated tumors was lower in the rMETase group [47].

In the present study, we tested a PDOX nude mouse model of BRAF V600E melanoma for sensitivity to rMETase in combination with TEM.

## RESULTS AND DISCUSSION

**Figure 1:**
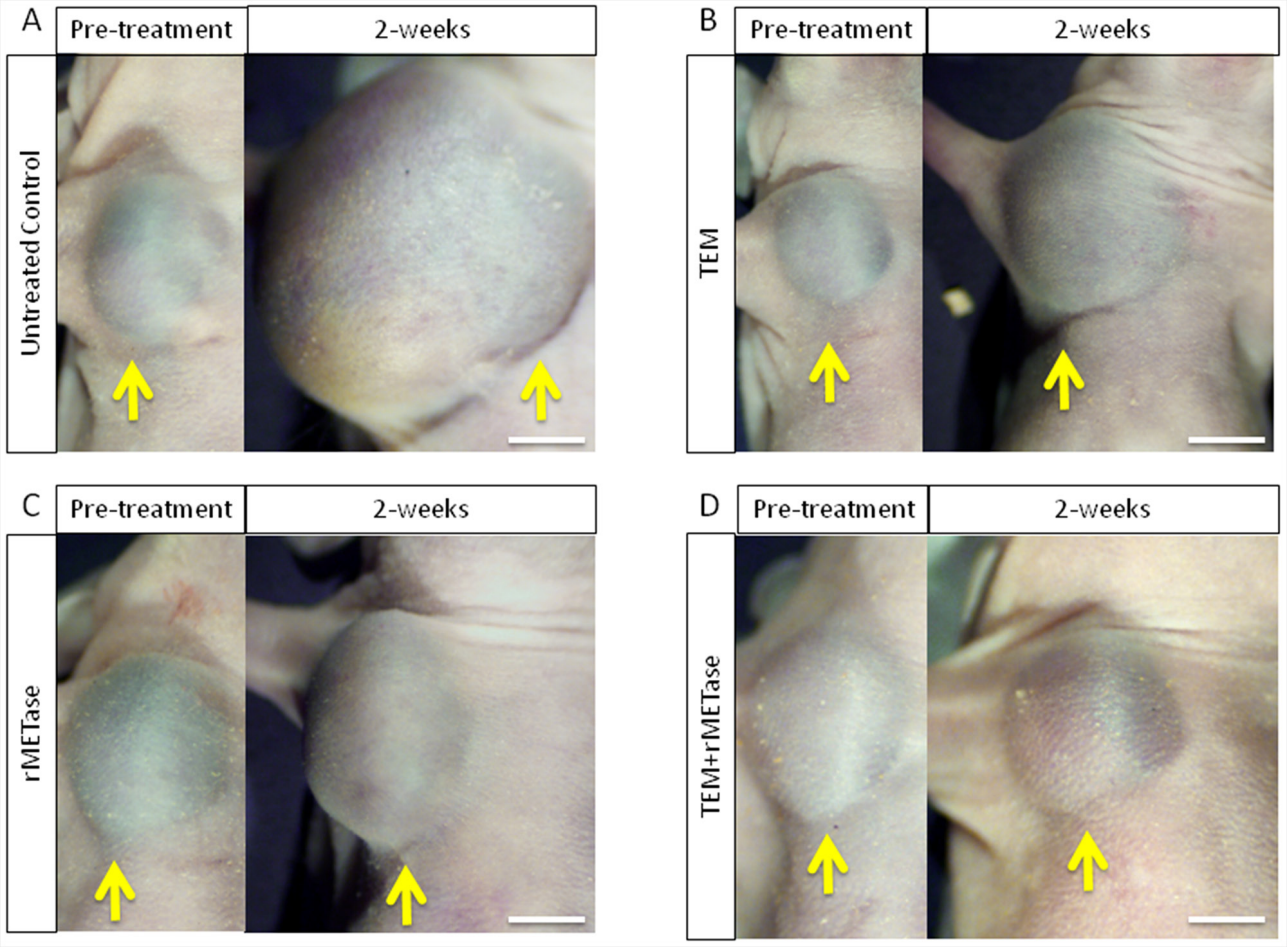
Macroscopic evaluation of therapeutic efficacy of TEM, rMETase and their combination on a BRAF V600E mutant melanoma **(A)** Tumor in untreated control. **(B)** Temozolomide (TEM). **(C)** Recombinant methioninase (rMETase). **(D)** Combination of TEM and rMETase. Yellow arrows show PDOX tumor growing on right chest wall. Scale bar: 5 mm.

**Figure 2:**
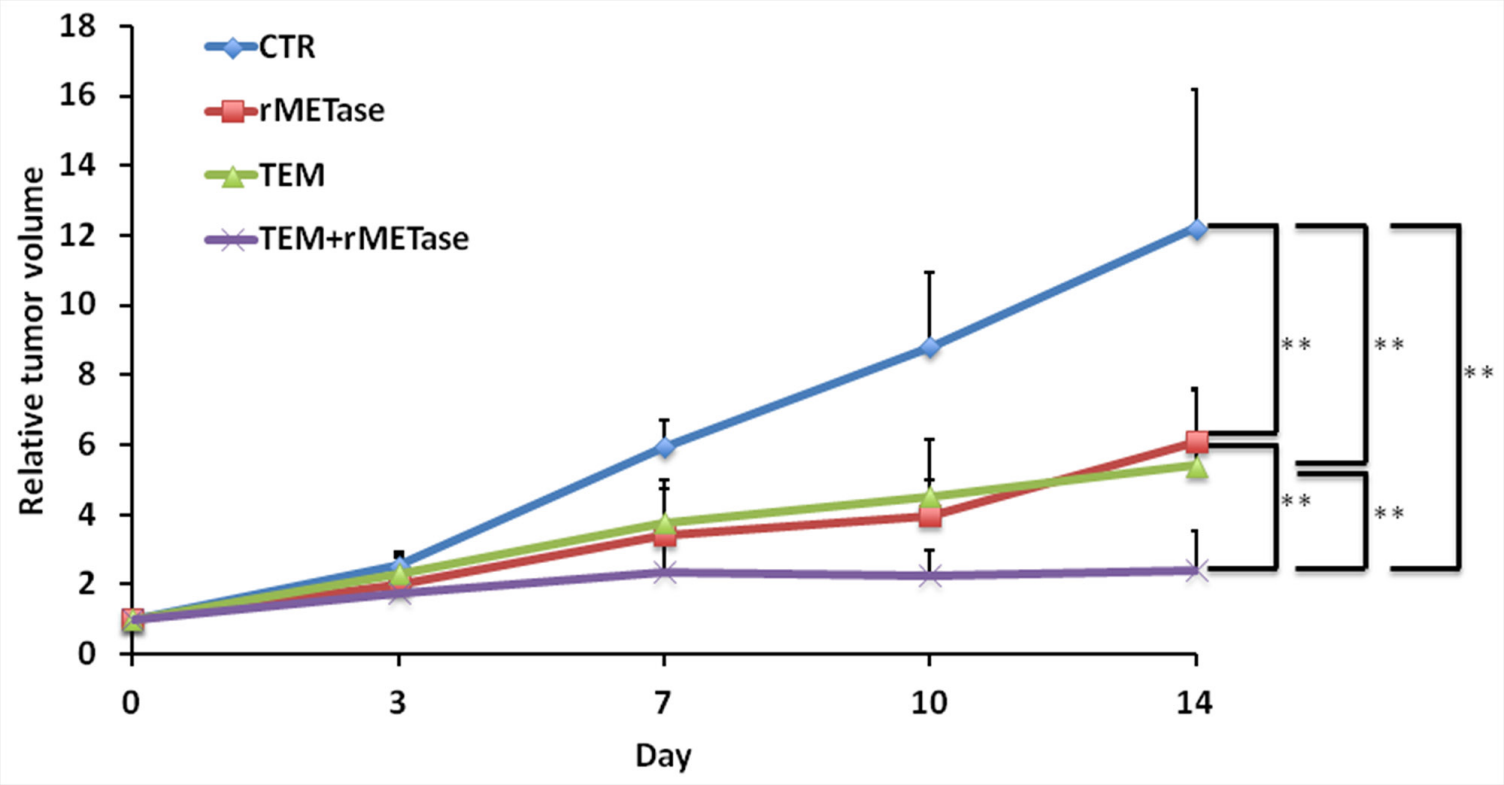
Time-coursed treatment efficacy of TEM, rMETase and their combination in the BRAF V600E mutant melanoma Line graphs show relative tumor volume at each time point relative to the initial tumor volume. All treatments significantly inhibited tumor growth compared to untreated control (TEM: *p*=0.0081; rMETase: *p*=0.0037; TEM-rMETase: *p*=0.0024). In addition, TEM and rMET combination therapy was significantly more efficacious than either TEM (*p*=0.0051) or rMETase (*p*=0.0051) alone at day 14. There was no significantly difference between TEM and rMETase. **p < 0.01. Error bars: ± SD.

All treatments inhibited tumor growth compared to untreated control (TEM: *p*=0.0081; rMETase: *p*=0.0037; TEM-rMETase: *p*=0.0024) on day 14 after initiation. Combination therapy of TEM and rMETase had significantly better efficacy than either therapy alone (TEM: *p*=0.0051, rMETase: *p*=0.0051). There was no significant difference between TEM and rMETase monotherapy (*p* =0.1282) (Figures 1 and 2).

**Figure 3:**
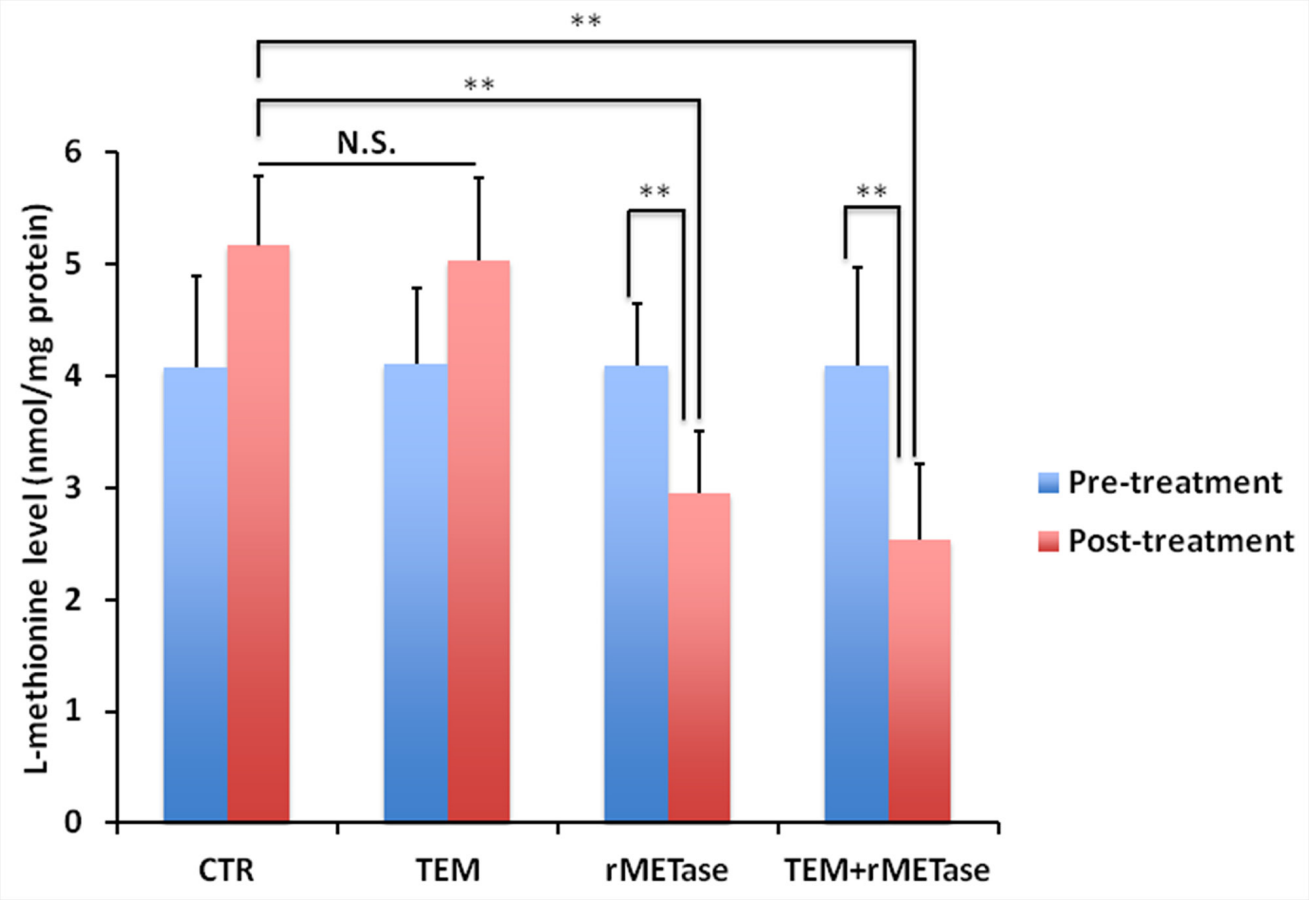
Intra-tumor L-methionine levels after rMETase treatment Bar graphs show L-methionine levels in each treatment group at rMETase or TEM pre- and post-treatment. rMETase significantly decreased intra-tumor L-methionine level. **p < 0.01.

Post-treatment L-methionine levels in tumors treated with rMETase alone or along with TEM significantly decreased compared to untreated control (*p* < 0.0001) (Figure 3). These results showed that the BRAF-V600E mutant melanoma PDOX is MET dependent and rMETase thereby suppresses its growth. The results also show that TEM similarly suppressed the melanoma PDOX. Future experiments will determine if there are any similarities in the mechanism of tumor inhibition of the two therapeutics.

**Figure 4:**
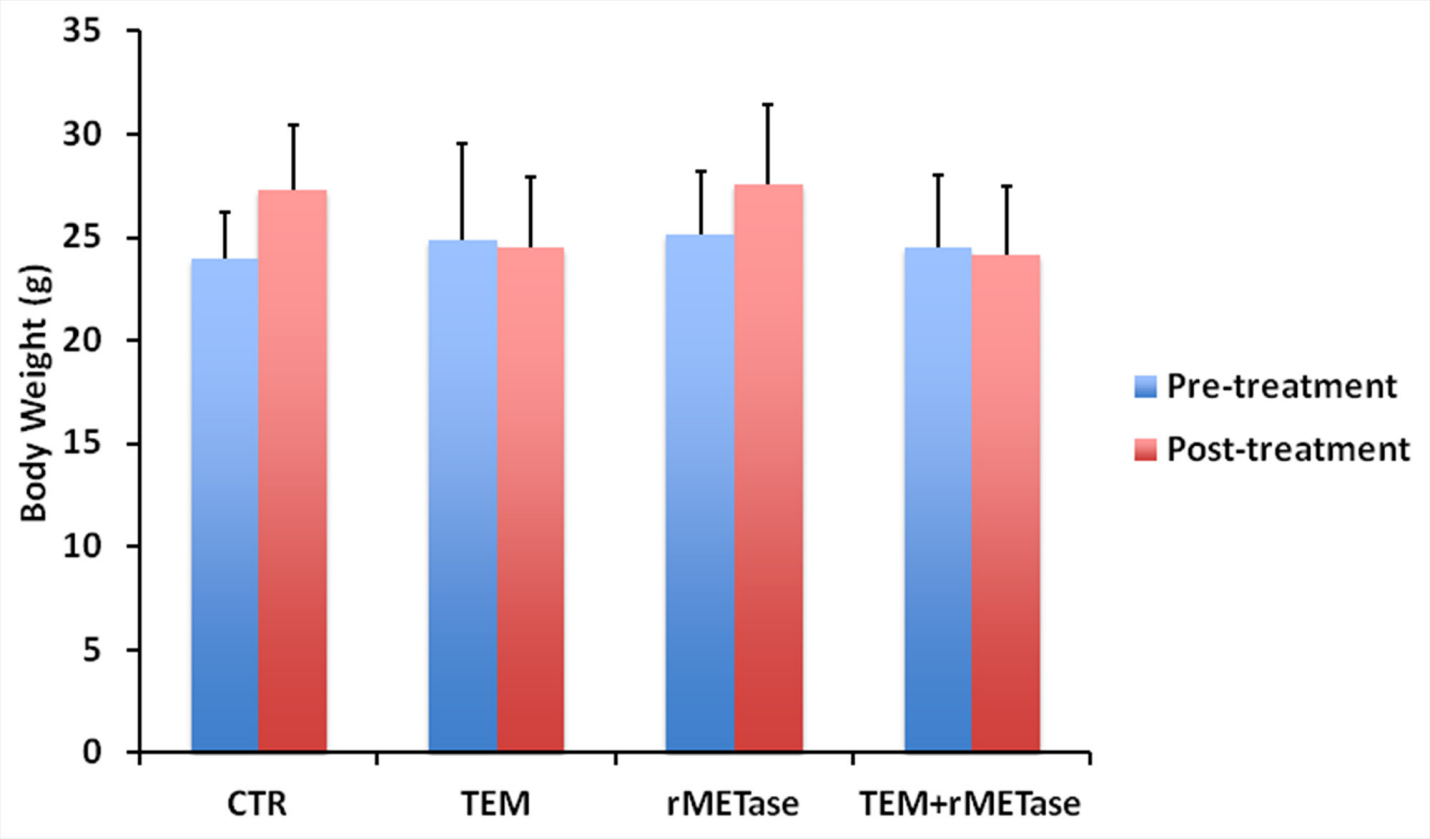
Effect of rMETase or TEM on mouse body weight Bar graphs show mouse body weight in each treatment group at pre- and post-treatment.

Body weight loss was observed only in the treatment groups including TEM. rMETase alone did not cause body weight loss (Figure 4). There were no animal deaths in any group.

**Figure 5:**
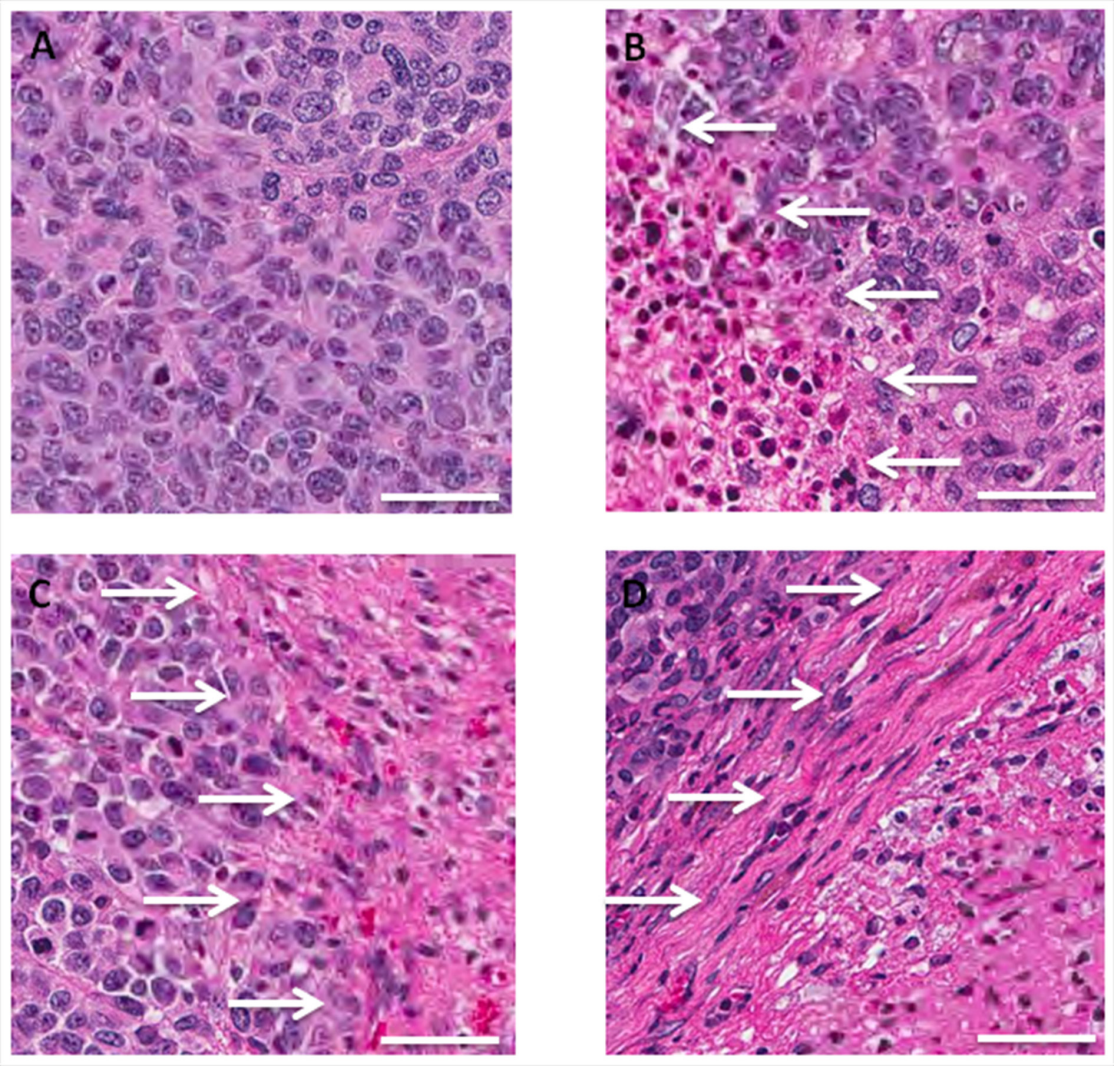
Tumor histology in untreated and TEM and rMETase-treated BRAF-V600E mutant melanoma PDOX models **(A)** Untreated control was comprised of viable cells without obvious necrosis. Epithelioid melanoma cells, devoid of melanin, with a high mitotic index are present. **(B)** Tumor treated with TEM showed partial necrosis. **(C)** Tumor treated with rMETase. Mitotic figures are present, indicating rMETase did not completely arrest the cell cycle. Tumor treated with rMETase showed partial necrosis similar to TEM. **(D)** Tumor treated with the combination of TEM and rMETase showed extensive necrosis. White allows: necrotic areas. Scale bars: 50 μm.

Histologically, the untreated control tumor was mainly comprised of viable cells. Epithelioid melanoma cells, devoid of melanin, with a high mitotic index, were observed [26]. In the tumors treated with rMETase only, there were still mitotic figures present indicating that rMETase did not completely arrest the tumor. The same degree of necrosis was observed in tumors treated with TEM and rMETase as monotherapy. Tumors treated with the combination of TEM and rMETase showed extensive necrosis, suggesting tumor necrosis is a major pathway of tumor growth arrest, but apoptosis may play a role as well (Figure 5).

TEM is a first-line chemotherapy for melanoma; however, with limited response. The present study has important implications since this is the first *in vivo* efficacy study of rMETase combination therapy on a patient tumor, in this case, a PDOX model of melanoma, a recalcitrant cancer. We had previously demonstrated that rMETase potentiates TEM in a mouse model of a human glioma cell line [46].

The first hint that methionine metabolism is perturbed in cancer came almost 60 years ago when Sugimura et al. [48] observed that rat tumor growth was slowed by giving the rats a defined diet depleted in methionine. Approximately 45 years ago, it was observed that L5178Y mouse leukemia cells in culture required very high levels of methionine to proliferate [49]. Subsequently, most cancer cell lines were found to be methionine dependence [50, 51]. These cell lines were derived from various cancer types including liver, pancreatic ovarian, submaxillary, brain, lung, bladder, prostate, breast, kidney, cervical, colon, fibrosarcoma, osteosarcoma, rhabdomyosarcoma, leiomyosarcoma, neuroblastoma, glioblastoma and melanoma. The occurrence of methionine dependence among these diverse cancer types suggests that methionine dependence may be a general phenomena in cancer. Normal unestablished cell strains, thus far characterized, grow well in methionine-depleted medium [50].

Human patient tumors, including tumors of the colon, breast, ovary, prostate, and a melanoma, were also found to be methionine dependent in Gelfoam® histoculture [52].

For more about metabolic disturbances in melanoma, please see Slominski et al. [53].

Cell cycle analysis demonstrated that the cells are arrested in the S/G_2_ phases of the cell cycle upon methionine restriction [42, 43, 52, 54, 55]. This is in contrast to a G_1_-phase accumulation of cells, which occurs only in methionine-supplemented medium at very high cell densities and is similar to the G_1_ block seen in cultures of normal fibroblasts at high density.

Recently, a paper appeared with the title “The new anticancer era: tumor metabolism targeting” [56]. This “new anticancer era” started in 1959 with the observation of Sugimura et al. [48] that depriving animals of methionine arrested tumor growth. It is our hope that this era will continue and lead to more effective cancer treatment, especially for recalcitrant cancers such as melanoma.

## MATERIALS AND METHODS

### Mice

Athymic *nu/nu* nude mice (AntiCancer Inc., San Diego, CA), 4–6 weeks old, were used in this study. Mice were housed in a barrier facility in a high efficacy particulate arrestance (HEPA)-filtered rack under standard conditions of 12-hour light/dark cycles. The animals were fed an autoclaved laboratory rodent diet. All animal studies were conducted in accordance with the principles and procedures outlined in the National Institutes of Health Guide for the Care and Use of Animals under Assurance Number A3873-1. All mouse surgical procedures and imaging were performed with the animals anesthetized by subcutaneous injection of a ketamine mixture (0.02 ml solution of 20 mg/kg ketamine, 15.2 mg/kg xylazine, and 0.48 mg/kg acepromazine maleate). The response of animals during surgery was monitored to ensure adequate depth of anesthesia. The animals were observed on a daily basis and humanely sacrificed by CO_2_ inhalation if they met the following humane endpoint criteria: severe tumor burden (more than 20 mm in diameter), prostration, significant body-weight loss, difficulty breathing, rotational motion and body temperature drop.

### Patient-derived tumor

A 75-year-old female patient was previously diagnosed with a BRAF-V600E melanoma of the right chest wall. The tumor was previously resected in the Department of Surgery, University of California, Los Angeles (UCLA). Written informed consent was provided by the patient, and the Institutional Review Board (IRB) of UCLA approved this experiment [24-26].

### Establishment of PDOX models of melanoma by surgical orthotopic implantation (SOI)

Subcutaneously-grown melanoma was harvested and cut into small fragments (3 mm^3^). After nude mice were anesthetized with the ketamine solution described above, a 5-mm skin incision was made on the right chest into the chest wall, which was split to make space for the melanoma tissue fragment. A single tumor fragment was implanted orthotopically into the space to establish the PDOX model. The wound was closed with a 6-0 nylon suture (Ethilon, Ethicon, Inc., NJ, USA) [24-26].

### Recombinant methionase (rMETase) production

Recombinant L-metionine α-deamino-γ-mercapto-methane lyase (recombinant methioninase, [rMETase]) [EC 4.4.1.11] from *Pseudomonas putida* has been previously cloned and was produced in *Escherichia coli* (AntiCancer, Inc.,). rMETase is a homotetrameric PLP enzyme of 172-kDa molecular mass [39].

### Treatment study design in the PDOX model of melanoma

PDOX mouse models were randomized into four groups of 10 mice each: untreated control (n=10); TEM (25 mg/kg, oral [p.o.], 14 consecutive days, n=10); rMETase (100 units, intraperitoneal [i.p.], 14 consecutive days, n=10); TEM + rMETase (TEM: 25 mg/kg, p.o., rMETase: 100 units, i.p., 14 consecutive days, n=10). Tumor length and width were measured both pre- and post-treatment. Tumor volume was calculated with the following formula: Tumor volume (mm^3^) = length (mm) × width (mm) × width (mm) × 1/2. Data are presented as mean ± SD. The tumor volume ratio is defined at the tumor volume at post-treatment time point relative to pre-treatment tumor volume.

### Imaging of the melanoma PDOX model

Imaging of the macroscopic tumor was performed with the OV100 Small Animal Imaging System (Olympus, Tokyo, Japan) [63].

### Intra-tumor L-methionine level analysis

Each tumor was sonicated for 30 seconds on ice and centrifuged at 12,000 rpm for 10 minutes. Supernatants were collected and protein levels were measured using the Coomassie Protein Assay Kit (Thermo Scientific, Rockford, IL). Protein levels were calculated from a standard curve obtained with a protein standard, bovine serum albumin (BSA). L-methionine levels were determined with the HPLC procedure described previously [47, 64]. Standardized L-methionine levels were calculated per mg tumor protein.

### Histological examination

Fresh tumor samples were fixed in 10% formalin and embedded in paraffin before sectioning and staining. Tissue sections (5 μm) were deparaffinized in xylene and rehydrated in an ethanol series. Hematoxylin and eosin (H&E) staining was performed according to standard protocols. Histological examination was performed with a BHS System Microscope (Olympus Corporation, Tokyo, Japan). Images were acquired with INFINITY ANALYZE software (Lumenera Corporation, Ottawa, Canada) [24-26].

### Statistical analysis

JMP version 11.0 was used for all statistical analyses. Significant differences for continuous variables were determined using the Mann-Whitney *U* test. Line graphs expressed average values and error bars show SD. A probability value of *P* ≤ 0.05 was considered statistically significant [24-26].

## CONCLUSIONS

The present study has demonstrated high efficacy of rMETase in combination with TEM in a BRAF-V600E mutant melanoma PDOX model. This is the first report to our knowledge in which rMETase combination therapy was tested on a patient-derived tumor in a mouse model. These results indicate the potential of rMETase combination therapy in the clinic and demonstrate the powerful precision of the PDOX model to identify active drugs and combination therapy on recalcitrant cancer.

Previously-developed concepts and strategies of highly-selective tumor targeting can take advantage of molecular targeting of tumors, including tissue-selective therapy which focuses on unique differences between normal and tumor tissues [57-62].
